# Image-guided LINAC radiosurgery in hypothalamic hamartomas

**DOI:** 10.3389/fneur.2022.909829

**Published:** 2022-09-01

**Authors:** Pantaleo Romanelli, Francesco Tuniz, Sara Fabbro, Giancarlo Beltramo, Alfredo Conti

**Affiliations:** ^1^Cyberknife Center, Italian Diagnostic Center (CDI), Milan, Italy; ^2^Department of Neurosurgery, ASUFC “Santa Maria della Misericordia”, Udine, Italy; ^3^Dipartimento di Scienze Biomediche e Neuromotorie (DIBINEM), IRCCS Istituto delle Scienze Neurologiche di Bologna, Alma Mater Studiorum-Università di Bologna, Bologna, Italy

**Keywords:** gelastic seizures, hypothalamic hamartoma, image-guidance, LINAC, stereotactic radiosurgery, multidrug-refractory epilepsy

## Abstract

**Introduction:**

Hypothalamic hamartomas (HH) are developmental malformations that are associated with mild to severe drug-refractory epilepsy. Stereotactic radiosurgery (SRS) is an emerging non-invasive option for the treatment of small and medium-sized HH, providing good seizure outcomes without neurological complications. Here, we report our experience treating HH with frameless LINAC SRS.

**Materials and methods:**

We retrospectively collected clinical and neuroradiological data of ten subjects with HH-related epilepsy that underwent frameless image-guided SRS.

**Results:**

All patients underwent single-fraction SRS using a mean prescribed dose of 16.27 Gy (range 16–18 Gy). The median prescription isodose was 79% (range 65–81 Gy). The mean target volume was 0.64 cc (range 0.26–1.16 cc). Eight patients experienced complete or near complete seizure freedom (Engel class I and II). Five patients achieved complete seizure control within 4 to 18 months after the treatment. Four patients achieved Engel class II outcome, with stable results. One patient had a reduction of seizure burden superior to 50% (Engel class III). One patient had no benefit at all (Engel class IV) and refused further treatments. Overall, at the last follow-up, three patients experience class I, five class II, one class III and one class IV outcome. No neurological complications were reported.

**Conclusions:**

Frameless LINAC SRS provides good seizure and long-term neuropsychosocial outcome, without the risks of neurological complications inherently associated with microsurgical resection.

## Introduction

Hypothalamic hamartomas (HH) are epileptogenic developmental malformations, growing inside the hypothalamus (1). They can be classified as sessile (or intrahypothalamic) or pedunculated (or parahypothalamic), if the HH grows within the third ventricle (1). Their size is commonly less than 2 cm, but larger or even giant lesions can be found as well (1). Unlike other brain tumors that induce an epileptogenic activity because of mass effect or brain edema, HH-neurons are characterized by an intrinsic epileptogenic activity, generating severe and medically-refractory seizures, with long-term neuropsychological sequelae (1–8). In particular, it has been suggested that neuronal gap junctions between small GABAergic HH-neurons contribute to epileptogenesis generating synchronous activity within the neuronal networks in HH tissue (1–8).

The mammillothalamic tracts are often compressed and distorted by the HH and mediate seizure spreading toward the anterior thalamus and cortex (2). Early seizure onset in newborns and childhood is often associated with multidrug-refractory epilepsy leading to a wide spectrum of cognitive delay and behavioral deterioration (1, 2, 7, 8). Developmental delay is not uncommon, when seizures are uncontrolled (3). Gelastic seizures, generalized seizures and drop attacks are common (1, 3). Early-onset seizures are poorly responsive to medical therapy, requiring timely surgical or radiosurgical intervention to prevent severe neuropsychological sequelae (3, 7, 8). A milder clinical course is associated with late seizure onset (2, 3, 7, 8). Surgical approaches include microsurgical resection through the transcallosal interforniceal, pterional or subfrontal translamina terminalis routes, microsurgical disconnection, endoscopic resection or disconnection, radiofrequency ablation, laser thermal ablation, and interstitial brachytherapy (9–13). Stereotactic radiosurgery (SRS) is an emerging non-invasive option for the treatment of small and medium-sized HH, providing excellent seizure outcomes without neurological sequelae (3, 4, 14). Image-guided frameless SRS delivering 6MV photon beams to the target in a non-isocentric fashion through a robotic linear accelerator (LINAC) has recently been reported as an option of treatment (3), and provides the least invasive stereotactic radiosurgical modality available, with proven submillimetric accuracy (3).

Here, we summarize our experience in a cohort of ten patients that underwent CyberKnife treatment, focusing on the role of SRS for HH-related epilepsy control and the appropriate timing for treatment delivery.

## Materials and methods

From January 2007 to December 2021, ten patients with HH-related pharmacologically-uncontrolled epilepsy underwent CyberKnife radiosurgery (Accuray Incorporated, Sunnyvale, CA). The same neurosurgeon (PR) performed all the procedures. Clinical assessment, endocrinological investigations and SRS results were retrospectively reviewed. Our Institutional Review Board approved the study, and informed consent was obtained from each patient.

The mean follow-up occurred at 118.2 ± 49.3 months (range 18–180). No subject was lost during the follow-up.

**Figure 1:**
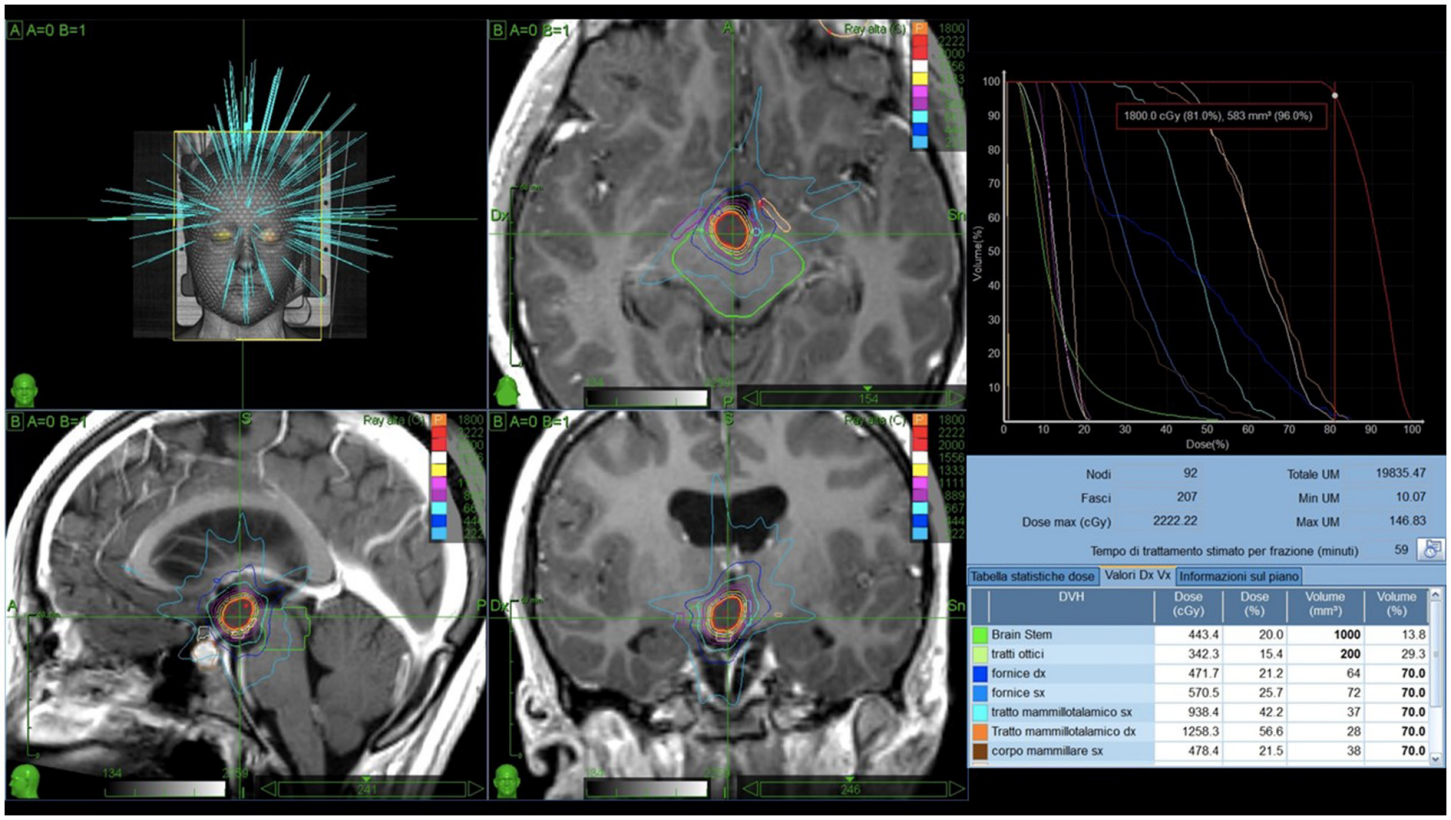
Example of treatment plan. A 3D simulation of the beams pathway delivering 18 Gy prescribed to the 81% isodose and the isodose curves on axial, sagittal and coronal T1-weighted scans are reported in **(A)**. **(B)** Shows the dose-volume histogram (DVH). Green: brainstem (443.4cGy, 20.0%). Light green: optic tracts (342.3cGy, 15.4%). Blue: right fornix (471.7cGy, 21.2%). Light blue: left fornix (570.5cGy, 25.7%). Azure: left mammillothalamic tract (938.4cGy, 42.2%). Orange: right mammillothalamic tract (1258.3cGy, 56.6%). Brown: left mammillary body (478.4cGy, 21.5%).

Basic demographic data on age and sex were recorded at the point of referral. No patient presented with precocious puberty or other endocrinological disorders. In all cases, antiepileptic treatment was unable to control seizures. All patients underwent preoperative magnetic resonance imaging (MRI) without and with contrast enhancement for SRS planning. CyberKnife stereotactic irradiation was delivered non-isocentrically to the HH as visible in T1-and T2-weighted volumetric MRI. Thin-cut computed tomography (CT) was fused with the MRI and used for intraoperative localization. Digitally reconstructed scans were fused with intraoperative digital X-ray scans providing the spatial reference frame needed for the accurate beam delivery by the robotic LINAC. Frameless single-session image-guided robotic radiosurgery was then performed. Nearby critical structures included the optic chiasm, pituitary gland, brainstem, mammillary bodies, mammillothalamic tract, and fornices. The dose delivered to the optic chiasm was kept below 5 Gy. Figure 1 reports an example of treatment plan.

Patients underwent serial clinical and neuroradiological investigations (brain MRI without and with contrast administration), starting 6 months after the treatment to assess the presence of radio-induced edema or radionecrosis. Engel classification was used to assess seizure control. Seizure freedom was classified as absence of partial and generalized seizures. Clinical deterioration, neurological status, perilesional radio-induced edema, radionecrosis and HH dimensional modifications were recorded.

## Results

Ten patients underwent single-staged image-guided frameless LINAC SRS. Table 1 summarizes their characteristics, while Table 2 depicts Engel class modifications during the follow-up. The population was composed of six males and four females, with a mean age of 26.1 ± 14.7 years (range 8–53). All patients, except one (who needed re-irradiation), underwent single-fraction SRS using a median prescribed dose of 16 Gy (mean 16.27 Gy). The mean prescription isodose was 76% (median 79%). The median target volume was 0.61 cc (mean 0.64 cc). The mean average dose delivered to the target was 19.02 Gy (median 19.32 Gy). The mean maximum dose was 21.53 Gy (median 20.51 Gy).

**Table 1:** Clinic characteristics of the population.

**Patient**	**Sex**	**Age**	**Volume**	**Prescribed**	**Prescribed**	**Max dose**	**Mean dose**	***n***	**Time from seizure**	**Type of**	**Engel**	**Follow-up**
**ID**		**(years)**	**(mm^3^)**	**dose (Gy)**	**isodose (%)**	**(Gy)**	**(Gy)**	**beams**	**onset to treatment**	**seizures**	**outcome**	**(months)**
1	M	8	1,160	16	65	24.60	20.30	151	7	GS, MDGS	I	180
2	M	9	890	16	70	22.85	19.40	151	6	GS, MDGS	I	180
3	F	14	450	16	65	24.60	19.40	175	12	MDCPS, MDGS, RGS	IV	168
4	M	40	750	16	79	20.25	18.05	140	31	MDGS, RGS, DA	II	132
5	M	22	364	17	78	21.79	19.32	83	16	MDCPS, MDGS, RGS	III	
5	M	24	567	16	80	20.00	18.00	211	18	MDGS, rare GS	III	120
6	F	22	256	16	79	20.25	18.07	80	18	frequent CPS, occasional GS	II	120
7	M	31	755	16	78	20.51	18.31	91	22	MDCPS, MDGS, RGS	II	108
8	F	15	607	18	81	22.22	20.00	207	8	MDGS, rare GS	I	96
9	M	53	555	16	80	20.00	18.40	113	46	Dacrystic seizures, MDCPS, RGS	II	60
10	F	49	737	16	81	19.75	19.93	143	41	MDGS, RGS	II	18
Mean		26.1	644.6	16.27	76.00	21.53	19.02	140.45	2.5			118.2
SD		14.7	241.4	0.62	5.92	1.74	0.83	143.68	12.9			49.3
Median		22.0	607.0	16.00	79.00	20.51	19.32	143.00	18.0			120.0

CPS, complex partial seizures; DA, drop attacks; GS, generalized seizures; MDCPS, multiple daily complex partial seizures; MDGS, multiple daily gelastic seizures; RGS, repeated (2–4 month) generalized seizures.

**Table 2:** Engel class evolution.

**Patient ID**	**0 months**	**3 months**	**6 months**	**12 months**	**18 months**	**24 months**	**30 months**	**36 months**	**>36 months**
*1*	4	4	4	2	1	1	1	1	1
*2*	4	4	4	2	1	1	1	1	1
*3*	4	4	4	4	4	4	4	4	4
*4*	4	4	4	4	3	2	2	2	2
*5*	4	4	1	1	1	3*	3	3	3
*6*	4	4	2	2	2	3	2	2	2
*7*	4	4	4	2	2	2	2	2	2
*8*	4	4	1	1	1	1	1	1	1
*9*	4	4	4	3	2	1	1	3	2
*10*	4	4	4	3	2				

Here we graphically represent Engel outcome of each patient. Red: Engel class IV. Orange: Engel class III. Yellow: Engel class II. Green: Engel class I. *: retreatment.

Currently, eight patients out of ten experience complete (Engel class I, three patients) or near complete (Engel class II, five subjects) seizure freedom, one patient has a reduction of seizure burden superior to 50% (Engel class III) and one subject has no benefit at all (Engel class IV). Five patients achieved complete seizure control within 4 to 18 months after the treatment (average age 13.5 years; average time from epileptic onset to treatment 6.5 years). Two patients classified as a class I after treatments experienced seizure relapse. In one case, seizure control failed after 14 months. Twenty-one months after the first treatment, the patient underwent SRS and achieved class III outcome. The other one relapsed to class III 2 year after the treatment. Adjustment of the medical therapy guaranteed a stable class II outcome.

The youngest patients of the cohort experienced the most satisfactory outcomes. The complete and long-lasting seizure control of an 8 and 9-year-old males, both affected by multidrug-refractory epilepsy, is reported in a previous report (3). Another young patient, a 14-year-old female, experienced complete seizure remission, with the exception of occasional prodromes of gelastic seizure without the subsequent crisis (“pressure to laugh”) (15).

Five patients achieved Engel class II outcome, with stable results (average age 40.7 years; average time from severe seizure onset to treatment 32.7 years). Clinical improvement required a long time (range: 12–36 months) in this group. One subject developed Engel class III after being temporarily classified in class I. One patient experienced no seizure improvement (Engel class IV outcome) and refused further treatments. No neurological complication has been found. Follow-up MRI (at 6, 12 and 24 months) showed the absence of perilesional radio-induced edema or radionecrosis and no (6 cases) or mild (≤3 mm, 4 cases) shrinkage of the lesion.

Of note a comparison between the Engel class I group and Engel class II group of patients showed a statistically significant difference (albeit the numerosity of the cohorts is limited) in terms of age of treatment 10.6 vs. 39.4 years (*p* < 0.0081 t-Student test) and seizure onset to treatment time 7 vs. 31.6 years (*p* < 0,0138 t-Student test).

## Discussion

HH are developmental malformations usually characterized by a relatively small intrahypothalamic lesion generating a severe epileptic encephalopathy (7, 8). While mass effect is relatively rare, severe drug-refractory seizures are rather common (2, 5, 7, 8, 10, 14). Stereo-EEG recordings showing that ictal onset was located inside the HH have been crucial to affirm the role of the hypothalamic lesion as the epileptogenic focus (16) and to direct the therapeutic efforts toward the hypothalamic lesion (17). Further evidence regarding the role of HH in the generation of seizures has been provided by the electrophysiological study of slices obtained from surgical specimens showing an intrinsic epileptogenic activity, characterized by the predominance of small GABAergic inhibitory neurons with an intrinsic “pacemaker-like” behavior (1).

Gelastic seizures are the hallmark of HH (18–20), but this tumor can induce dacrystic seizures, complex partial seizures, generalized tonic or tonic-clonic seizures and drop attacks (1–6). While gelastic and dacrystic seizures originate within the HH, complex and generalized seizures could be ascribable to secondary spreading through the mammillothalamic tracts (2).

Early onset in childhood can be associated with an epileptic encephalopathy resembling the Lennox-Gastaut syndrome (2, 3, 5, 6, 17). Findings of temporal and frontal localization of ictal and interictal EEG epileptic activity promoted unsuccessful frontal and/or temporal lobectomies (17). The demonstration of an intrinsic epileptogenic activity of HH (16) allowed to direct the therapeutic efforts toward the resection or ablation of the HH itself (5, 6, 16, 17).

Direct HH resection, disconnection or ablation are effective in improving seizure control (4, 9–14). The improvement in seizure control is linked to the extent of the surgical intervention (4). Subtotal resection, disconnection, or ablation is associated with incomplete seizure control, while seizure freedom can be induced by a more aggressive approach (4).

A variety of surgical routes and approaches have been developed over the last decades (3). Resective surgery is an excellent option for large pedunculated HH with limited hypothalamic attachment (and consequent reduced chance to develop metabolic complications) (7, 8, 11, 17). However, open surgery remains bound to the risk of neurological sequelae arising from vascular or hypothalamic damage, such as thalamo-capsular infarcts resulting in hemiparesis or hemiplegia, oculomotor palsy, visual field deficits, short-term memory deterioration, hyperphagia, hypothyroidism, and diabetes insipidus (3, 7, 8, 11, 17). Complete seizure freedom is difficult to achieve but remarkable long-term improvement of disabling seizures has been reported (7, 8, 11, 17). Seizure recurrence requiring further intervention is relatively common (4). Minimally invasive surgical approaches, including endoscopic resection/disconnection and radiofrequency/laser ablation, have been preferred to open surgery because of a lower morbidity, but long-term seizure control remains elusive (9, 10, 12, 13, 21). Interstitial brachytherapy, with the stereotactic implantation of radioactive I^125^ seeds inside the HH (22), appeared as a promising option decades ago but has now fallen out of favor due to the less invasive and greater conformal dose distribution provided by radiosurgery (3, 4).

SRS is an emerging treatment for HH either as primary option or as a second treatment in patients with residual HH and recurrent seizures (3, 4). The majority of epileptogenic HH are small intrahypothalamic or medium-sized sessile intraventricular/interpeduncular lesions (3, 4). SRS provides an excellent approach to treat these lesions, which are hard to resect without causing major neuro-metabolic injury (3, 4). The mechanism of action of SRS responsible for the seizure control is unknown: the lack of target necrosis as shown by follow-up MRI points toward a neuromodulatory effect induced by gliosis, down-regulation of firing neurons and reduced vascular supply (3, 23, 24).

The presence of delicate anatomic structures adjacent to HH (hypothalamic nuclei, mammillary bodies, fornices, mammillothalamic tracts, optic chiasm, optic tracts and brainstem) requires extremely careful surgical and radiosurgical planning (3). Size, location and symptomatology of HH are crucial factors driving the choice to deliver either surgical or radiosurgical treatments (3). Pedunculated HH growing inside the interpeduncular fossa enter in close spatial relationship with the optic chiasm anteriorly, the optic tracts sideways and the brainstem posteriorly (1, 2). They are typically larger lesions, usually associated with endocrine dysfunction and/or mass effect symptoms but not with epilepsy (1, 2). Pedunculated HH are thus more amenable to microsurgical or endoscopic resection (1, 2, 4). Intrahypothalamic hamartomas are located within the wall of the third ventricle between the post-commissural fornix anteriorly, the mammillothalamic tract posteriorly, and the mammillary body inferiorly (1, 2). Small unilateral sessile HH have been widely described as epileptogenic (1, 2, 5, 6, 25, 26). Due to their relatively small size and intrahypothalamic location, SRS is considered as a valuable option for their treatment, inducing poikilothermia in rare cases (14). SRS has been shown to be not only safe but also effective in controlling gelastic and generalized seizures originating from sessile HH (4, 14, 26).

Successful radiosurgical treatment of epileptogenic HH was first reported in 1998: a non-enhancing 10 mm-diameter spherical lesion, that was located on the floor of the third ventricle, was treated using GammaKnife with a marginal dose of 18 Gy (26). After a temporary increase in seizure frequency, the patients became seizure free and the 12-months follow-up MRI demonstrated the complete disappearance of the tumor (26).

In a cohort of 57 patients with HH-induced drug-refractory epilepsy and severe cognitive and psychiatric comorbidities, at 3-years follow-up, Régis reported an Engel class I outcome rate of 39.6%, Engel class II of 29.2% and Engel class III of 20% (14). Twenty-eight patients required a second treatment (14). A complete or near-complete seizure control was achieved in 68.8% of the population (14). The median frequency of seizure was 107.3 seizures per month before radiosurgery, 16 seizures per month at 3 years, and 7 seizures per month at last follow-up (14). In patients experiencing seizure cessation, the median delay was 30 months with a minimum of 4 months and a maximum of 139 months (14). Global psychiatric comorbidity improved in 56% and remained stable in 28% of the cohort (14). No permanent neurological side effect was reported on the long-term follow-up, while a temporary seizure worsening in the first week after the procedure in cases of prescribed doses >16 Gy and transient non-disabling poikilothermia were described (14).

Based on this experience, a prospective multicenter study was conducted. The preliminary results on a cohort of ten patients with medically-refractory epilepsy that underwent GammaKnife radiosurgery were reported (23). The mean marginal dose was 15.5 Gy (range 12–20 Gy) and the median maximal diameter of the HH was 13.5 mm (range 8–22 mm) (23). The mean volume of the marginal isodose was 889.4 mm3 (range 134–2674.8 mm3) (23). The main challenge in SRS planning was the proximity of the lesion to the optic pathways and the hypothalamus (23). To guarantee a maximum dose of 10 Gy to these structures, in some cases the HH was undercovered (23). All patients had improved seizure control after radiosurgery, with four patients seizure free (Engel class I), two patients with infrequent seizures (Engel class II), and two with reductions in frequency but persistence of occasional generalized seizures (Engel class III) (23). Two subjects experienced unsatisfactory seizure control after the first GammaKnife radiosurgery and became seizure free after a second treatment (23). This study suggested an association between efficacy and dose: the marginal dose was more than 17 Gy for all patients in the successful group and <13 Gy for all subjects in the improved group (23). Substantial behavioral improvement was noticed in two cases (23). No side effect was reported (23). Follow-up MRI showed no perilesional edema and shrinkage of the lesion in two patients while no change of size was detected in the others (23).

The update of this report described a 60-patients cohort (24). At a 3-years follow-up, seizure freedom and persistence of non-disabling seizures was found in 40 and 20% of the population, respectively (24). No permanent neurological complication was noted and sleep quality, behavioral and learning performance improvements were reported (24).

The presence of a dose effect, with an interdependence between seizure control and marginal dose, was confirmed by other small clinical series (27–30). After 12–68 months from the delivery of doses of 12–14 Gy, a decrease in seizure frequency and intensity was reported, but no patient became seizure free (28, 29). Barajas (27) reported substantial improvement in seizure control following treatment in 3 patients receiving 12.5, 14, and 15 Gy. Tonic-clonic seizures disappeared completely after 8–12 months, whereas gelastic seizures disappeared almost completely in 2 patients (27). Dunoyer (30) described a 4 and 5-year children with medically refractory seizures associated with HH, that were treated with GammKnife radiosurgery, delivering 11 Gy to the 85% isodose and 14 Gy to the 45% isodose. The latter patient became seizure free, whereas the former experienced a substantial reduction in seizure frequency (24, 25). Overall, it appears that doses in the range of 12–14 Gy may result in relief from seizures, but the degree of amelioration is variable: early treatment may be associated with more favorable outcomes and could allow the use of lower doses (27–31), as in our cohort. Delivery of high doses is not uniformly associated with excellent seizure outcomes: in a small group of 4 patients with a long history of symptoms (range 4–28 years), only modest improvements were achieved after the delivery of 17.5 Gy (31).

The eligibility of patients for radiosurgical treatment of HH depends mainly on the combination of anatomical and dosimetric factors, such as the volume of the lesion, the presence of nearby radiosensitive structures, and the dose required to achieve the therapeutic goal. The treatment goal of radiosurgery for HH is to deliver doses high enough to affect epileptogenesis without exceeding the tolerance of nearby critical structures (4). Moreover, the two largest HH series measured, respectively, a median lesional size of 15 mm (5) and a mean size of 19 mm (2). The smallest lesions were entirely or predominantly intraventricular, whereas the larger lesions were both intraventricular and interpeduncular (2). Radiosurgical treatment can be performed safely on HH with sizes ranging close to the above-mentioned measurements. These lesion volumes allow steep radiosurgical dose gradients providing relatively high doses to the HH while the adjacent critical structures receive much lower and well-tolerated doses. No serious permanent complications have been reported after radiosurgery. A case of severe radiation-induced edema requiring long-term steroid administration despite a relatively low-dose (13 Gy prescribed to the 85% isodose line) has been also described (32).

Concerning the use of SRS devices different from GammaKnife, De Salles et al. (33) studied the efficacy of LINAC radiosurgery on gelastic seizures. Of the three patients that were treated with doses of 15–18 Gy, two became seizure-free 7 and 9 months after radiosurgery, and the third experienced a substantial reduction in seizure frequency (class II) (33).

Frameless image-guided LINAC radiosurgery is a novel option for the treatment of HH, providing a non-invasive treatment without sacrificing the submillimetric accuracy of SRS (3, 4). The absence of a stereotactic frame provides greater comfort for younger patients and opens up a wide additional space for beam trajectories, extending the range of beam penetrations to the splancnocranium and consequently enhancing the beam access to skull base or deep brain lesions (4).

Image-guided frameless robotic radiosurgery using CyberKnife for the treatment of HH has been previously described (3, 4). It was recently reported that early treatment was associated with favorable outcomes for children with multidrug-refractory epilepsy: long-term seizure freedom and major neuropsychological improvements without complications have been achieved in two patients (8 and 9 years old) undergoing early treatment (3).

In our series, all patients underwent single-fraction SRS using a median marginal dose of 16 Gy, with a median prescription isodose of 79%. The median volume of the target was 0.61 cc. The majority of patients experienced complete or near complete seizure freedom. Three subjects achieved Engel class I seizure-control within 4 to 18 months after treatment. Two more patients experienced temporary seizure relief and subsequent seizure relapse within 2 years: one underwent re-irradiation, achieving Engel class III outcome, while the other one is currently in class II. Other four patients achieved Engel class II outcome, with stable results. One patient has a reduction of seizure burden superior to 50%, while one subject experienced no seizure improvement. This failure is likely explained by the presence of a small intrahypothalamic post-surgical residual not included in the target volume.

Moreover, despite the limited numerosity of our casuistry, we can underline a link between seizure control and early treatment: patients with decades of persisting seizures are likely to develop secondary epileptogenesis, leading to partial or complete failure of the treatment. However, it's never too late: two of the oldest patients are currently in class II.

In agreement with previous series (3, 4), our experience confirms that the best candidates for radiosurgery are patients with small, intrahypothalamic hamartomas, while larger pedunculated lesions are preferably treated by resection of the intraventricular part, eventually followed by radiosurgery on the intrahypothalamic residual. Moreover, young patients with a short seizure history reach seizure freedom, while older patients with a long epileptic history improve in seizure control without complete seizure disappearance, suggesting that appropriate timing is essential to maximize results (3, 7, 8, 18).

The delayed efficacy is a limit of SRS: several months are needed to achieve seizure improvement or complete control. In patients with very severe epilepsy deteriorating actively over time, it may be a clinical issue. Under these circumstances, resection or treatment options with more immediate efficacy might be a better option.

Management of the patients who fail to respond to radiosurgery remains a difficult issue and is likely related to secondary wide-spread epileptogenesis. The literature shows that all the surgical techniques reach about the same probability of 60% of seizure freedom (7, 8, 11, 17). Thus, whatever the surgical technique, 40% of the patients get a disappointing result. SRS should always be considered after surgical failure. A repeated radiosurgical treatment can also be considered for patients with unsatisfactory seizure control after a primary radiosurgical failure or after seizure relapse following an interval of effective seizure control (3, 4).

In conclusion, this report outlines the result of the largest cohort of HH-patients treated with frameless LINAC SRS. It confirms that frameless robotics SRS appears to be a safe and effective non-invasive treatment for medically-refractory epilepsy induced by HH. Small intrahypothalamic lesions in young patients with a short seizure history respond well to SRS. Early treatment is associated with excellent long-term prognosis in children with medically-refractory epilepsy. Timing of the treatment is of paramount importance to prevent cognitive decline due to uncontrolled seizures.

## Data availability statement

The original contributions presented in the study are included in the article/supplementary material, further inquiries can be directed to the corresponding author.

## Ethics statement

Our Institutional Review Board (CDI-Milan) approved the study, and informed consent was obtained from each patient. Written informed consent to participate in this study was provided by the participants' legal guardian/next of kin. Written informed consent was obtained from the individual(s), and minor(s)' legal guardian/next of kin, for the publication of any potentially identifiable images or data included in this article.

## Author contributions

PR, FT, and SF: first draft, editing, review, and final approval. GB and AC: review and final approval. All authors contributed to the article and approved the submitted version.

## Conflict of interest

The authors declare that the research was conducted in the absence of any commercial or financial relationships that could be construed as a potential conflict of interest.

## Publisher's note

All claims expressed in this article are solely those of the authors and do not necessarily represent those of their affiliated organizations, or those of the publisher, the editors and the reviewers. Any product that may be evaluated in this article, or claim that may be made by its manufacturer, is not guaranteed or endorsed by the publisher.
